# Folate receptors and transporters: biological role and diagnostic/therapeutic targets in cancer and other diseases

**DOI:** 10.1186/s13046-019-1123-1

**Published:** 2019-03-12

**Authors:** Barbara Frigerio, Claudia Bizzoni, Gerrit Jansen, Christopher P. Leamon, Godefridus J. Peters, Philip S. Low, Larry H. Matherly, Mariangela Figini

**Affiliations:** 1 Dipartimento di Ricerca Applicata e Sviluppo Tecnologico, Fondazione IRCCS Istituto Nazionale dei Tumori, Milan, Italy; 2 Amsterdam Rheumatology and Immunology Center, Amsterdam University Medical Center, location Vrije Universiteit, Amsterdam, The Netherlands; 3 Endocyte Inc, West Lafayette, Indiana, USA; 4 Department of Medical Oncology, VU University Medical Center, Cancer Center Amsterdam, Amsterdam, The Netherlands; 5 Purdue University Institute for Drug Discovery, West Lafayette, Indiana, USA; 6 Barbara Ann Karmanos Cancer Institute and Wayne State University School of Medicine, Detroit, MI USA; 7 Present address: ASST Papa Giovanni XXIII, Bergamo, Italy

**Keywords:** Folate, Folate receptors, Folate transporters, Cancer targeting, Inflammation targeting, One-carbon metabolism, Imaging, CAR T cell

## Abstract

Folate receptors and transporters and one-carbon metabolism continue to be important areas of study given their essential roles in an assortment of diseases and as targets for treatment of cancer and inflammation. Reflecting this, every 2 years, the Folate Receptor Society organizes an international meeting, alternating between North America and Europe, where basic and translational scientists, clinical oncologists and rheumatologists from both academia and industry convene in an informal setting. The 7th International Symposium on Folate Receptors and Transporters was held in Sant’Alessio Siculo (ME), Taormina, Italy from 1st to 5th of October 2018, organized by Dr. Mariangela Figini from Fondazione IRCCS Istituto Nazionale dei Tumori, Milan. Following the format of previous meetings, more than 50 scientists from 9 different countries attended the 2018 meeting to share ongoing developments, discuss current research challenges and identify new avenues in basic and translational research. An important feature of this meeting was the participation of young investigators and trainees in this area, two (A. Dekhne and N. Verweij) of whom were awarded fellowships to attend this meeting as a recognition of the high scientific quality of their work. This report provides a synopsis of the highlights presented in the following sessions: Barton Kamen Lecture; Targeting one-carbon metabolism in cytosol and mitochondria; Structure and biology of the one-carbon solute transporters; Physiology and pathophysiology of folate receptors and transporters; Folate receptors for targeting tumors and inflammatory diseases; Conventional and new anti-folate drugs for treating inflammatory diseases and cancer; Imaging; Ongoing clinical trials; and Chimeric Antigen Receptor cell therapies of cancer.

## Barton Kamen lecture

**Fig. 1:**
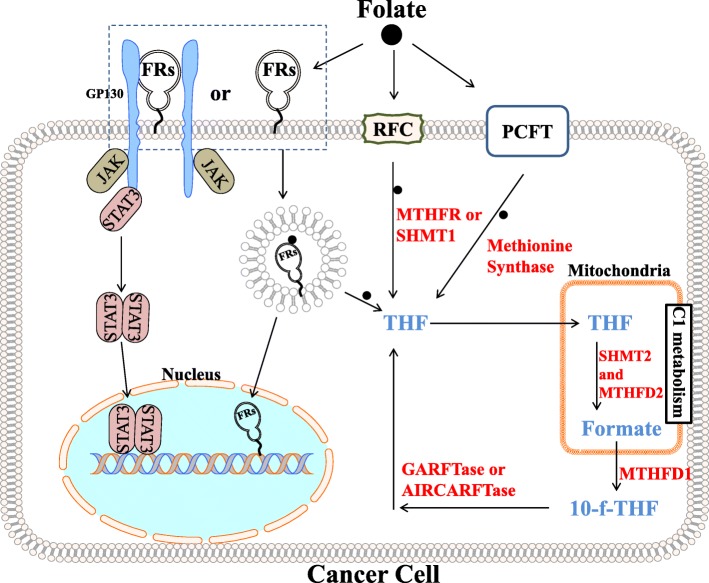
Main aspects of folate receptor signaling and C1 metabolism discussed during the meeting. Three types of folate transporters/receptors are known to exist in humans to facilitate the uptake of folate: Folate Receptors (FRs), Reduced Folate Carrier (RFC) and Proton-Coupled Folate Transporter (PCFT) [57]. Left side: Folate binding to FRs can induce STAT3 activation via a GP130 co-receptor mediated JAK-dependent process. Folate can also bind FRs undergoing endocytosis and upon released FRs are set free to act like transcription factors [22]. Right side: Folate, through an interlinked set of mitochondrial and cytosolic reactions, support the C1 metabolism and the main pathway reactions are depicted [1, 6]. THF: tetrahydrofolate; MTHFR, methylenetetrahydrofolate reductase; SHMT1/2, serine hydroxymethyl transferase in cytosol (1) and mitochondrial (2); MTHFD1, methylenetetrahydrofolate dehydrogenase 1; MTHFD2, methylenetetrahydrofolate dehydrogenase 2; 10-f-THF: 10-formyl- tetrahydrofolate; GARFTase: glycinamide ribonucleotide formyltransferase; AICARFTase: 5-aminoimidazole-4-carboxamide ribonucleotide formyltransferase

Professor **Joseph Bertino** (Cancer Institute of New Jersey, Rutgers University, USA) gave the lecture *“The mitochondrial folate enzyme MTHFD2 as a target for anticancer therapy”* in honor of the late Barton Kamen, a pioneering physician-scientist and major contributor to folate receptor (FR) biology for many decades. Dr. Kamen was a former trainee during his pediatric residency at Yale and a later colleague at the Cancer Institute of New Jersey and the Robert Wood Johnson Medical School, now part of Rutgers University. Dr. Bertino gave an overview of one-carbon (C1) metabolism, its compartmentalization in mitochondria and cytosol, and the promise of therapeutic targeting mitochondrial C1 metabolism in cancer. Mitochondrial C1 metabolism, provides glycine, NAD(P) H, ATP and C1 units for cytosolic biosynthetic reactions [1]. Key enzymes in this pathway include serine hydroxymethyltransferase (SHMT) 2 and NAD-dependent methylene tetrahydrofolate dehydrogenase (MTHFD) 2, both of which are frequently upregulated in cancer [2]. Dr. Bertino noted that like certain other C1 enzymes, MTHFD2 has been reported in the nucleus where it co-localizes with DNA replication sites [3]. The significance of this finding is still evolving. Moreover, adult and embryonic tissues express mitochondrial MTHFD2L, a bifunctional enzyme, homologous to MTHFD2, utilizing NAD/NADP as cofactor [4]. In his talk, he noted that MTHFD2 is highly expressed in rapidly replicating tumor cells but not in normal adult tissues, providing a strong rationale for targeting this enzyme for selective cancer treatment (see Fig. 1 right side). He noted that MTHFD2 is a bifunctional enzyme with both MTHFD and cyclohydrolase activities that distinguish it from the homologous trifunctional cytoplasmic enzyme MTHFD1 that includes dehydrogenase, cyclohydrolase and formyltetrahydrofolate synthetase activities. Bertino described approaches for the rational design of MTHFD2 inhibitors, drawing from structural studies of homologous enzymes including the human cytoplasmic MTHFD1 for which the bifunctional methylenetetrahydrofolate dehydrogenase/cyclohydrolase domain has been crystalized with an inhibitor (LY345899). Although no inhibitor for MTHFD2 has yet emerged, its overexpression in rapidly replicating tumor tissues but not in normal tissues and the finding that knockdown of MTHFD2 effects a strong antiproliferative response in tumor cells provide compelling rationale for targeting MTHFD2 in cancer [5, 6].

## Targeting one-carbon metabolism in cytosol and mitochondria

Following Bertino’s lecture, several speakers further discussed the subject of compartmentalization and targeting C1 metabolism. **G. Ducker** (University of Utah, USA) gave a presentation entitled *“Isotope tracing of compartmentalized folate metabolism”* in which he described analytical methods for characterizing the sub-cellular compartmentalization of C1 metabolism including the metabolic fluxes. The focus was on mass spectrometry-based isotope tracing techniques that have enabled determination of the precise localization of folate reactions within cells and have supported development of novel inhibitors of mitochondrial and cytosolic C1 reactions in cancer [7].

A particular focus of several presentations was on the mitochondrial C1 metabolism enzyme, SHMT2, the first enzyme in serine catabolism that is induced upon hypoxic stress in Myc-transformed cells [8]. Expression of SHMT2 is highly correlated with the malignant phenotype across a broad spectrum of cancers, including lung, colon, breast, glioma, and liver [2]. Despite the unequivocal importance of SHMT2 to development and maintenance of the oncogenic phenotype, there are no clinically relevant inhibitors of this important target. **A. Gangjee** (Duquesne University, USA) presented the work *“Design, synthesis and evaluation of first-in-class SHMT inhibitors”* in which he described the design, molecular modeling, and synthesis of “first-in-class” 5-substituted pyrrolo [2,3-*d*] pyrimidine compounds AGF291, AGF320, and AGF347 that inhibit the mitochondrial “oncodriver” SHMT2, along with cytosolic SHMT1, and C1 purine biosynthetic enzymes, glycinamide ribonucleotide formyltransferase (GARFTase) and/or 5-aminoimidazole-4-carboxamide ribonucleotide formyltransferase (AICARFTase) [9]. **L. Matherly** (from the Barbara Ann Karmanos Cancer Institute, USA) presented “*Discovery of novel pyrrolopyrimidine compounds that inhibit mitochondrial and cytosolic one-carbon metabolism with potent anti-tumor efficacy*”, demonstrating the potential of these novel compounds as broad-spectrum antitumor agents toward lung, colon and pancreatic tumors [10]. Antitumor efficacy reflected inhibition at mitochondrial C1 metabolism at SHMT2, and at cytosolic targets including C1-dependent purine biosynthesis and SHMT1, detected by targeted metabolomics with stable isotope tracers. Promising in vivo anti-tumor efficacy with curative potential was demonstrated in an aggressive pancreas cancer model. **C. Dann III** (Indiana University, USA) presented the *“Biochemical and structural analysis of targeted cytotoxic therapeutics on folate utilizing enzymes in C1 metabolism”* in which he described the enzymology and structural biology of target enzymes including GARFTase, AICARFTase/inosine monophosphate cyclohydrolase (ATIC), SHMT1, SHMT2 and MTHFD2 with the lead pyrrolo [2,3-*d*] pyrimidine inhibitors [11]. His presentation described efforts to determine crystal structures of these enzyme inhibitor complexes to facilitate future drug design. Disparate activities of the lead molecules as cell-based inhibitors vis á vis isolated enzymes suggested an importance of cellular transport and polyglutamylation. Finally, **A. Dekhne** (Barbara Ann Karmanos Cancer Institute, USA) confirmed for the lead compound AGF347 (following radiolabeling) uptake by the reduced folate carrier (RFC), proton-coupled folate transporter (PCFT) and folate receptor (FR) α, and mitochondrial accumulation and metabolism to AGF347 polyglutamates in both cytosol and mitochondria. Collectively, these studies establish the pyrrolo [2,3-*d*] pyrimidine analog AGF347 as an exciting prototype for multi-targeting mitochondrial and cytosolic C1 metabolism for cancer, with substantial promise for overcoming resistance to current anticancer therapies [12].

## Structure and biology of the one-carbon solute transporters

Presentations by **M. Jansen** (Texas Tech University Health Sciences Center, USA) and **Z. Hou** (Barbara Ann Karmanos Cancer Institute, USA) focused on the major folate transport system including the facilitative transporters PCFT and RFC, and FRα. Both PCFT and FRα have been a focus for targeted drug delivery [10, 13], whereas RFC is the major tissue folate transporter for physiologic folates and clinically important antifolates such as methotrexate. The presentation by M. Jansen on the *“Path to structural understanding of folate transport through PCFT”* was aimed to understand PCFT function and determination of its atomic structure and molecular mechanism of transport. These approaches require large amounts of functional protein and several high-level heterologous expression systems were used for human PCFT including insect cells and yeast [14]. PCFT was functional in whole cell assays in both expression systems and after solubilization, purification to homogeneity, and reconstitution into liposomes. High-throughput crystallization trials identified conditions for PCFT crystallization that were systematically optimized to yield crystals suitable for high-resolution structure determination. The availability of a robust overexpression system for human PCFT provides a basis for future biochemical, biophysical and structural studies of this physiologically and pharmacologically important transporter.

Hou presented the *“Impact of folate transport redundancies on cancer therapy with targeted and untargeted antifolates”.* The focus of his presentation was on functional inter-relationships (e.g., redundancies) among PCFT, FRs and RFC for novel tumor-targeted folate analogs (including those previously synthesized and studied by Gangjee and Matherly) such as AGF94 (a 6-substituted pyrrolo [2,3-*d*] pyrimidine inhibitor transported by both PCFT and FRs but not RFC), AGF102 (FR-selective 6-substituted thienoyl [2,3-*d*] pyrimidine analog, without substrate activity by PCFT or RFC), and classical antifolate drugs [pemetrexed (PMX), methotrexate (MTX)] that are variably transported by FRα, PCFT and RFC [15]. Towards this goal, he described cell line models using a PCFT-, FR-, and RFC-null HeLa background engineered to express FRα or RFC under control of a tetracycline (Tet)-inducible promoter without or with constitutive expression of PCFT. Studies of transporter expression and function over a range of induction demonstrated that co-expression of the major folate transporters can result in transporter redundancies or antagonism with variable and surprisingly disparate impacts on anti-tumor efficacies of both classical and tumor-targeted antifolates depending on their transport specificities. The results identified critical determinants of anti-tumor activity with targeted and untargeted antifolates, including levels of transporter expression, intracellular folates, as well as extracellular pH.

## Physiology and pathophysiology of folate receptors and transporters

A number of presentations covered various aspects of the physiology and pathophysiology of FR and facilitative transporters. Professor **Satyajit Mayor** (National Centre for Biological Sciences, Bangalore, India) opened this session with the keynote lecture “*Role of folate receptors in the organization of membranes of eukaryotic cells”*. Since 1994 Dr. Mayor focused his interest on FRs on the basis of the evidences provided by Kamen about intracellular FR trafficking [16]. Mayor has focused his work on FRs as prototypical glycosylphosphatidyl inositol (GPI)-anchored proteins with the goal of defining the role of the local structure and composition of the outer membrane in the control of membrane processes and mechanisms such as signaling, sorting, and exo- and endocytic processes. In his lecture, Mayor gave an overview of the physiology and pathophysiology of FRs and covered the mechanisms behind GPI-anchored proteins endocytosis and nanoclustering. Endocytic processing of GPI-anchored proteins, wherein they are preferentially endocytosed via a clathrin and dynamin-independent endocytic pathway, results in their uptake into an extremely acidic early endosomal compartment [17]. This is likely to be important for folate uptake. The unusual organization of the FR at the cell surface has sparked a new understanding of the specialized membrane microenvironment necessary for their function, where small nanoclusters of GPI-anchored proteins in a “sea” of monomers are generated in response to specific signals [18]. Detailed understanding of the physico-chemical principles behind the generation of these nanoclusters via the active mechanics of actin filaments and myosin is emerging. Studies of organization and trafficking of the GPI-anchored human FR provide unique insights into the endocytic pathways available at the cell surface to delineate a complex picture of membrane organization and how these machineries may be relevant for specific-FR functions and for their exploitation in imaging and targeted therapy [19, 20].

**P. Martensen** (Aarhus University, Denmark) gave a presentation *“Folate receptor alpha and STAT3 activation”* that extended her previous in vitro observations that folic acid can activate STAT3 through FRα in a Janus Kinase (JAK)-dependent manner, and that gp130 functions as a transducing receptor for this signaling in the FRα-expressing tumor cells [21] (see Fig. 1, left side). Martensen also showed that breast tumors in female PyMT mice receiving a high folic acid diet displayed significantly increased tumour volumes along with STAT3 activation, compared to mice receiving a normal diet. Moreover, FRα knockdown abolished folic acid-induced STAT3 activation [22].

**J. Kaur** (Institute of Medical Education and Research, India) described in the presentation “*Multiple Regulatory Mechanisms Control the Expression of Folate Transporters in Conditions of Ethanol Exposure and Folate Deficiency”* her research on the regulation of the major folate transport systems in response to folate deficiency [23]. Folate deficiency can occur in both developing and well-developed countries with inadequate dietary folate intake and severe alcoholism as the most frequent causes. In a rat model of chronic ethanol feeding, folate transport decreased, accompanied by decreased expression of PCFT, RFC and FR in intestine, liver, colon and kidney, while increased mRNA expression of PCFT and RFC was recorded in pancreas. PCFT and RFC associated with membrane lipid rafts were decreased under conditions of chronic alcoholism. In response to dietary folate deficiency in rats, there was a significant decrease in folate levels, accompanied with increased uptake of folic acid across the membranes in intestinal and liver tissue and increased expression of folate transporters and FRs. A better understanding of the biological basis for physiologic folate deficiency in response to limiting dietary folate and chronic alcoholism will identify strategies for addressing this condition.

Folates are essential for brain development and function. Brain folate uptake primarily occurs at the choroid plexus through the concerted action of the FRα and PCFT. Inactivating mutations on FRα or PCFT cause cerebral folate deficiency, resulting in childhood neurodegeneration. The presentation by **R. Bendayan** (University of Toronto, Canada) “*Up-regulation of reduced folate carrier by vitamin D enhances folate uptake at the blood-brain barrier”* elaborated on the functional expression of RFC in brain microvessel endothelial cells representative of the blood-brain barrier (BBB) and its upregulation by the vitamin D nuclear receptor (VDR). The detection of RFC functional expression in various in vitro and in vivo BBB model systems suggests a potential role for this transporter in mediating brain folate permeability, especially when the predominant route of folate uptake at the choroid plexus is impaired. These data are the first to show that activation of VDR through calcitriol treatment can upregulate RFC functional expression at the BBB, implying that this transporter could potentially constitute a novel strategy for brain folate delivery and treating neurometabolic disorders caused by folate deficiency [24].

The regulation of folate transport in Cerebral Folate Deficiency (CFD) was the focus of the studies presented by **R. Finnell** and **R. Cabrera** (both from Center for Precision Environmental Health, USA) on *“DNA binding transcriptional repression and the regulation of folate transport”.* CFD syndrome is defined as any neurological syndrome associated with low concentrations of 5-methyltetrahydrofolate in cerebrospinal fluid, while folate levels in plasma and red blood cells are within normal range. Previously, mutations in several folate pathway genes, including FRα, dihydrofolate reductase, and PCFT were identified in CFD patients [25]. This now includes 70 individuals diagnosed with CFD who have been studied by genomic, whole exome sequencing and in vitro functional analyses. Clinical work-up of these patients failed to reveal any abnormalities in brain imaging but indicated disturbed cerebral folate transport. A de novo mutation in human Capicua gene (CIC), c.1057C > T (p.R353X), was identified in a patient that caused the protein to degrade through nonsense-mediated mRNA decay (NMD) such that less CIC protein is detected in the patient’s fibroblast and iPS cells. A CIC target binding octamer sequence occurs in the promoter regions of the folate transport genes FRα, PCFT, and RFC1, and dihydrofolate reductase, and binding was confirmed by chromatin immunoprecipitation assays. CIC conditional knockout mice showed similar phenotypes to CFD proband, including ASD-like behaviors and development delay. Thus, a fortuitous clinical interaction led to intensive mouse genetic studies that supported a causal interaction with CIC and CFD in a heretofore understudied regulatory mechanism of folate transport.

In cerebral folate deficiency folate transport across the choroid plexus is compromised. **R. Steinfeld** (University Children’s Hospital Zurich, Switzerland) discussed in the presentation *“Loss of folate receptor alpha function and cerebral folate deficiency”* the role of FRα in folate uptake across the choroid plexus and the transport of folates from the cerebral spinal fluid (CSF) into the brain parenchyma [26]. At the basolateral (blood-site) of choroid plexus epithelial cells, FRα binds folates, after which the receptor is endocytosed into multivesicular bodies from which FRα-containing vesicles/exosomes are formed and released in the CSF. From the CSF, FRα-containing vesicles/exosomes are taken up by ependymal cells by a putative FRα binding protein (i.e. Low Density Lipoprotein Receptor-Related Protein 2 (megalin)) on ependymal cells to deliver folates to astrocytes and neurons. A better understanding of the cell biological pathways and proteins involved in cerebral folate transport will lead to identification of the defects that contribute to CFD.

## Folate receptors for targeting tumors and inflammatory diseases

### Cancer

**Fig. 2:**
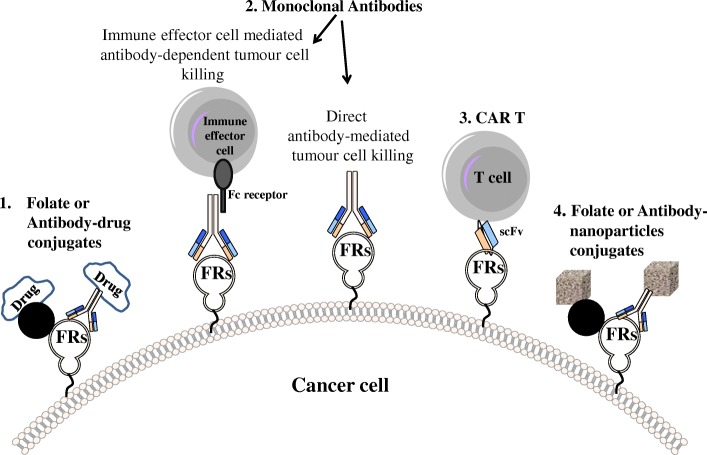
Approaches for targeting FRs as therapies for cancer and inflammatory diseases. 1) Folate [13] or Antibody [58] drug-conjugates [59], 2) Monoclonal antibodies (immune effector cell engagement): antibodies link FRs-expressing tumor cells with immune effector cells that bear Fc receptors leading to antibody-dependent effector cell-mediated cytotoxicity [27, 29], 3) Chimeric antigen receptor (CAR) T cells: CAR T cells recognizing FRs through scFv (single chain variable fragment) trigger tumor cell killing [60–62], 4) Folate or Antibody nanoparticles-conjugates [63]

Targeted therapies for cancer are aimed at maximizing tumor kill and minimizing toxicity. FRs were among the first validated targets for cancer since FRs are expressed in a subset of malignant cells and a limited number normal tissues, making FRs interesting Tumor Associated Antigens (TAAs) (see Fig. 2).

Monoclonal antibodies that target FRα on the surface of tumor cells are under evaluation in clinical trials [27–29]. They can mediate specific anti-tumor activity either by blocking cell signaling or by eliciting immune-mediated cell killing by engaging effector cells or complement. IgG antibodies are the commonly used class of antibodies in cancer therapy but other classes of antibodies are under evaluation. **S. Karagiannis** (King’s College, UK) investigated in her presentation *“Mechanisms of action for MOv18 IgE targeting folate receptor alpha-expressing tumours”* whether antibodies engineered with Fc regions of IgE class may provide an alternative approach for treating solid tumours. This concept is based on the properties of this class to mediate immune clearance of parasites by effector cells such as macrophages. Unlike commonly-used IgG1 and IgG4, the IgE Fc regions feature very high affinities for cognate Fcε-receptors on monocytes and macrophages, effector cells known to infiltrate tumours. Her group developed a first-in-class IgE antibody that recognizes FRα and exhibits superior tumour growth suppression compared with the corresponding IgG in two human tumour xenograft models. Efficacy was recapitulated in an immunocompetent syngeneic rat tumour model designed to more-closely mirror human IgE-FcεR interactions. These results provide critical support for clinical translation of IgE as an anti-cancer strategy, and facilitated the launch of a clinical trial of this agent for patients with ovarian carcinoma tumours. This strategy may offer opportunities to extend the current IgG-only class of monoclonal antibodies for the treatment of solid tumours [27].

Targeting of pharmaceuticals is a rapidly evolving strategy to overcome the difficulties in therapeutic delivery, especially to the tumor site. Unlike traditional drug delivery systems, nanoparticle-based compounds attain superior uptake by tumors via active or passive mechanisms. Several presentations highlighted different preparative techniques and types of nanoparticles for tumor targeting through folate.

**T. Pellegrino** (Italian Institute of Technology, Italy) described in the presentation *“Functionalized magnetic nanoparticles to target folate receptors to tackle cancer”* her studies with magnetic nanoparticles that act as heat mediators under oscillating magnetic fields in the so-called “magnetic hyperthermia”. The advent of non-hydrolytic methods for preparing of magnetic nanoparticles has resulted in better control in terms of size, size distribution and crystallinity (parameters affecting structural and magnetic properties, thus their heat performance). Pellegrino focused on her recent progress in use of cubic-shaped iron oxide magnetic nanoparticles as a delivery tool for heat-mediated drug delivery (i.e., synthesis, functionalization, characterization, and drug loading) and in vitro and in vivo (both subcutaneous and orthotopic models) studies with targeting magnetic nanoparticles to FRα-overexpressing ovarian cancer cells with anti-FRα antibody fragments [30]. Elemental and histological analyses showed that conjugated magnetic nanoparticles were specifically retained at tumor sites longer than the non-conjugated nanoparticles, suggesting their use as multifunctional theranostic agents [31].

**S. Biocca** (University of Roma “Tor Vergata”, Italy) in *“Selective targeting of folate-functionalized DNA nanocages”* described DNA-based nanostructures for delivering classical chemotherapeutic agents [32, 33]. DNA possesses intrinsic properties of high stability, biocompatibility and versatility that makes it an extremely suitable polymer for generating nanoparticles. Dr. Biocca’s group designed and assembled truncated DNA octahedral nanocages, functionalized with folate, to selectively target tumour cells overexpressing FRα. Folate-DNA nanocages are internalized in FR+ tumour cells with efficiencies greater than 40 times compared to cells not expressing FRs and are taken up in a time-dependent manner with high intracellular stability (> 48 h). While DNA nanocages are not themselves cytotoxic, they can be loaded with doxorubicin (DOX) and following delivery to the cytoplasm and DOX release, result in a cytotoxic response.

Failure of chemotherapy can be due to Multidrug Resistance (MDR), by which cancer cells develop resistance towards different chemotherapeutic drugs, often with unrelated structures and distinct pharmacodynamic properties. A major mechanism of MDR involves overexpression of P-glycoprotein (Pgp) which actively extrudes drugs. As an approach to bypass drug efflux by Pgp, **E. Gazzano** (University of Torino, Italy) during the *“Folate-targeted liposomal nitrooxy-doxorubicin: An effective tool against P-glycoprotein-positive and folate receptor-positive tumors”* talk presented data about the synthesis and validation of the efficacy of an innovative liposomal formulation which encapsulates a DOX-derivative conjugated with a nitric oxide releasing group. Liposomes were functionalized with folic acid as tumor targeting “antenna”, and their activity was evaluated in preclinical models of DOX-resistant breast tumors. This formulation revealed superior efficacy to DOX and Caelyx® (commercial DOX liposomal formulation), and showed a cardiovascular safety profile similar to Caelyx® [34, 35].

Folic acid conjugation to therapeutic molecules has been a bona fide approach for selective targeting of FR-positive cancer cells [36]. A poster presentation, “*Synthesis of N4Py Derivatives Containing Targeting Groups for Tumor Cells”*, by **R. de Vries** (University of Groningen, The Netherlands) described synthesis of a folic acid conjugate of a mimic of the anti-tumor drug Bleomycin (BLM), N,N-bis (2-pyridylmethyl)-N-bis (2-pyridyl)-methylamine (N4Py) [37]. One of the conjugates (N4Py-S-S-FA) contained a disulfide bond, which can be reduced intracellularly to facilitate release of N4Py. In vitro experiments demonstrated that N4Py-S-S-FA elicited good selectivity toward FRα-expressing KB and IGROV-1 tumor cells and is similarly cytotoxic as the parent iron complex of the ligand N4Py.

### Inflammation

Immune cells involved in inflammatory processes (e.g., tumor- associated macrophages (TAMs) and chronic inflammatory/autoimmune diseases (e.g. rheumatoid arthritis (RA)) express the FRβ isoform. FRβ expression is restricted to cells of the myeloid lineage, i.e., monocytes and macrophages. RA patients are characterized by synovial infiltration of FRβ-positive macrophages which can be imaged and/or targeted for therapy [38, 39]. Little is known about FRβ expression on macrophage precursors, i.e. circulating monocytes in peripheral blood. The presentation “FRβ expression profiles in RA blood and synovial tissue” by **G. Jansen** (VU University Medical Center, The Netherlands) showed work in progress indicating that in peripheral blood of RA patients, FRβ expression was highest on CD14 low/CD16 high subpopulation of monocytes.

#### Conventional and new anti-folate drugs for treating inflammatory diseases and cancer

Methotrexate (MTX) remains a first line of therapy for patients with rheumatoid arthritis (RA). However, despite its prominent use for more than 25 years, the mechanisms through which MTX suppresses inflammation in RA are not well understood. **A. Puig Kröger** (Instituto de Investigación Sanitaria Gregorio Marañón, Hospital General Universitario Gregorio Marañón, Spain) in *“Unmasking the anti-inflammatory effects of methotrexate in arthritis”* described the molecular impact of MTX on gene expression in inflammatory macrophages, a major cell type involved in the etiology of RA. In her presentation*,* Puig-Kröger demonstrated that low-dose MTX conditions macrophages towards a tolerant state by decreasing production of pro-inflammatory cytokines in response to pro-inflammatory stimulants (e.g. TNFα, LPS, RASF). MTX induces upregulation of the TNFAIP3 gene, which encodes for the protein A20, a negative regulator of NFκB signaling. By elevating expression of TNFAIP3, a gene already associated with reduced susceptibility to RA, the pro-inflammatory macrophage that is critical to maintenance of the inflammatory state in RA is reprogrammed to a quiescent or tolerant state. Studies were described showing 1) that knockdown of A20 strongly reduces the tolerance-inducing effect of MTX, and 2) that TNFAIP3 expression in peripheral blood cells is significantly higher in RA patients who respond to treatment with MTX than those who don’t. These studies establish a central role for upregulation of TNFAIP3 in the therapeutic mechanism of MTX in treatment of RA [40].

**R. de Jonge and I.B. Muller** (VU University Medical Center, The Netherlands) in their presentations *“Methotrexate TDM in arthritis”* described the design and optimization of a clinically applicable LC-MS/MS method for quantitation of methotrexate polyglutamate (MTX-PG) levels. They applied their method to monitor MTX-PG levels in three prospective cohort studies in adult and pediatric arthritis patients. Higher intracellular MTX-PGs were associated with improved clinical responses and inter-individual variations in MTX-PG levels were shown to be at least partly (20%) explained by age, baseline erythrocyte-folate, a single nucleotide polymorphism in the gene for folylpolyglutamate synthetase, and MTX dose [41].

**T. Ratliff’s** (Purdue University, USA) presentation “*Targeting Myeloid Cells in the Tumor Microenvironment”* described studies in mouse and human tumors to identify specific markers, including FRβ, in tumor-associated fibroblast and myeloid-derived suppressor cells, which feature an immune-suppressive phenotype. Targeting of these cells can be an attractive strategy to induce antitumor activity [42].

Presentations by **M.P. Costi** (University of Modena and Reggio Emilia, Italy), and **A.L. Jackman** and **U. Banerji** (The Institute of Cancer Research, UK) described studies on cancers treated with novel anti-folate drugs. In *“Dimer disrupters of thymidylate synthase (TS) through folate receptor targeting”* Dr. Costi described several compounds that interfered with the dimeric assembly of the homodimeric enzyme, TS, a major therapeutic target for antifolates and fluoropyrimidines. Inhibition of TS dimerization results in inhibition of enzyme activity, suggesting that these represent a new class of chemotherapy agents that interfere with folate metabolism.

In *“Preclinical and early clinical experience with BTG 945 (BGC945; ONX0801; CT900)”* Jackman and Banerji presented exciting preclinical and clinical data on a new antifolate BTG945 (ONX0801)*.* Recognizing that all FDA-approved antifolates enter cells via the RFC that is expressed in virtually all human cell types, and that FRα is expressed primarily on cancer cells with little-or-no expression on other human cell types except the proximal tubule cells of the kidneys, Jackman designed a new antifolate BTG945 that is transported into cells almost exclusively via FRα. Once internalized by tumours over-expressing FRα, BTG945 inhibits TS, resulting in anti-tumor activity. Preclinical pharmacokinetic and pharmacodynamic data defined the optimal blood drug levels for therapeutic efficacy in humans (12 mg/m^2^ every other week) and early clinical results demonstrated very promising activity in ovarian cancer patients with FRα positive tumours (46% of the patients showed a GCIG CA125 response and 70% displayed a Recist response). Ongoing trials are underway to establish the optimal dosing concentration and frequency with plans for a randomized Phase 2/3 study in the near future [43].

#### Imaging

Oncogene-driven deregulation of energy metabolism in malignant cells was recognized in 2011 as an emerging hallmark of cancer [44]. **F. Podo** (in quiescence Istituto Superiore di Sanità, Italy) gave a presentation entitled *“Cancer metabolic reprogramming: novel insights offered by molecular imaging”* that described molecular imaging approaches for in vivo monitoring of altered metabolic pathways in cancer cells. These include: 1) activation of aerobic glycolysis by [^18^F] fluorodeoxyglucose (FDG) positron emission tomography (PET) and hyperpolarized [^13^C] magnetic resonance spectroscopy (MRS)/magnetic resonance spectral imaging (MRSI); 2) deregulated phosphatidylcholine (PtdCho) cycle using [^1^H]MRS/MRSI and PET [^11^C]choline; and 3) the “flare” effect from folate-mediated TS inhibition on proliferation-dependent uptake of the PET imaging agent [^18^F]FLT. Her presentation focused on molecular imaging approaches to monitor folate-dependent C1 metabolism in vivo and comparison of folate radiotracers for PET imaging using different radionuclides (^18^F, ^68^Ga, ^44^Sc, ^152^Tb). She concluded that [^18^F] FLT PET is suitable for monitoring the effects of TS inhibitors in vivo, since it enables evaluation of the balance between the de novo and salvage pathways for generation of thymidylate [45, 46].

Presentations “*Preclinical FR-macrophage PET guided therapy response monitoring”* by **C. Molthoff** and *“[18F]Fluoro-PEG-Folate PET Imaging in Rheumatoid arthritis patients” by* **N. Verweij** (both from VU University Medical Center, The Netherlands) explored the utility of the PET tracer [^18^F]-fluoro-PEG-folate in quantifying the accumulation and retention of activated macrophages in the inflamed joints of patients with rheumatoid arthritis. Because cartilage degradation and bone erosion manifest in RA joints until successful treatment is initiated, early detection of RA is essential to prevent irreversible joint damage. Since activated macrophages constitute the major inflammatory cell type in RA lesions and these inflammatory macrophages (but not tissue resident macrophages in healthy tissues) express FRβ that can be imaged with [^18^F]Fluoro-PEG-Folate, the authors hypothesized that the intensity of an RA joint’s inflammation might be assessed by quantitating its uptake of the folate-targeted PET tracer mentioned above. Molthoff provided data from a rat model of RA demonstrating that uptake of [^18^F]Fluoro-PEG-Folate was significantly reduced in the inflamed joints following MTX therapy than before. This diminished accumulation of imaging agent was due to decreased number of activated macrophages in these joints. The FRβ + macrophage numbers were also reduced in the liver and spleen of RA rats following MTX therapy, suggesting that MTX suppressed the numbers of activated macrophages both locally (in inflamed joints) and systemically. Verweij evaluated the ability of [^18^F]Fluoro-PEG-Folate to image inflamed lesions in human patients with clinically diagnosed RA. Data from a phase 1 clinical trial demonstrated that the PET tracer specifically concentrated in the inflamed lesions of patients with RA, yielded better images of inflamed lesions in whole body scans better than the reference macrophage tracer because of a lower background signal and is safe for use in humans [47].

Another important use of folate for targeted imaging of FR-positive lesions was presented by **P. Low** (Purdue University, USA) in “*Tumor-targeted NIR dyes for fluorescence-guided surgery*”. With the goal of improving the ability to locate and completely remove malignant lesions during surgery, his research group developed tumor-targeted folate-targeted fluorescent dyes, a folate-fluorescein (EC17) and a folate-targeted near infrared dye (OTL38), that selectively bind to FRs on cancer cells and can be detected upon excitation with the proper light source. Within 2 h of intravenous injection, the targeted fluorescent dyes clear from healthy tissues and accumulate in malignant tissues, allowing facile distinction of tumor from adjacent healthy tissues. Results were presented for patients with lung, brain, kidney, ovarian and other cancers. Results presented by Low demonstrated that the targeted fluorescent dyes were highly specific for tumor tissue and that use of these dyes during surgery enabled surgeons to 1) find and remove more malignant lesions, positive tumor margins, malignant lymph nodes much more efficiently than was previously possible using standard surgical methods (i.e., palpation and visual inspection); 2) more accurately stage cancer patients. With the aid of new and improved NIR cameras currently under development, these tumor-targeted NIR dyes should significantly expand the tools available for fluorescence-guided surgery and increase the chances of complete tumor resection and consequent cancer patient survival [48].

#### Ongoing clinical trials

This session began with **Y. Setiady** (ImmunoGen, USA) presenting the *“Development of IMGN853, a folate receptor α (FRα) targeting antibody-drug conjugate (ADC), for ovarian cancer treatment”*. IMGN853 (mirvetuximab soravtansine) is an antibody-drug conjugate (ADC) comprised of the chimeric anti-FRα M9346A antibody, a humanized derivative of the previously described anti-FRa antibody Mov19 [49] linked to the tubulin-disrupting maytansinoid, DM4, via a sulfo-SPDB linker. IMGN853 binds to FRα on cancer cells and is subsequently internalized [50]; DM4 is released from the antibody through both enzymatic degradation and/or disulfide linker cleavage, resulting in disruption of cell division, followed by cell death. Phase 1 clinical data showed that IMGN853 has promising single-agent activity and a favorable safety profile against FRα-positive platinum-resistant ovarian cancer patients. IMGN853 is currently being evaluated in FORWARD I, a Phase 3 monotherapy study, as well as in combination with other agents (including pembrolizumab; anti-PD1 antibody) in a Phase 1b/2 study, called FORWARD II.

**C. Leamon** (Endocyte Inc., USA) followed with a presentation entitled *“Translational struggles of folate SMDCs: New learning and future directions”* in which he provided a summary spanning nearly 20 years of cancer-targeted small molecule drug conjugate research, with special emphasis on EC1456, a folate-tubulysin conjugate that was tested in a phase 1 trial [51]. Leamon reviewed a comprehensive dataset involving ovarian cancer lesion analysis, correlating immunohistochemical (IHC) staining results to the uptake of ^99m^Tc-etarfolatide (a SPECT-based imaging probe for functional FR expression) [52]. He also showed unpublished data on the quantization of EC1456 uptake, as well as associated metabolites in those same lesions.

As the role of biomarkers in drug discovery, development and clinical trials has gained increased importance there is great emphasis on quality assurance and assay validation so as to establish standardized guidelines for analytical methods used in biomarker measurements. The establishment of a concrete validation process that addresses technology, integration and method validation, as well as regulatory pathways for efficient biomarker development, is fundamental. In this context, **E. Somers** (Morphotek Inc., USA), **Y. Wettergren** (Institute of Clinical Sciences, Sweden) and **G. Peters** (VU University Medical Center, The Netherlands) described approaches for selecting patients for therapeutic trials, including the use of folate transporters such as FRα or PCFT as potential biomarkers. For instance, Somers described in “*Development and validation of the folate receptor IHC assay”* an IHC assay for selecting patients for inclusion in Morphotek’s therapeutic trials involving FRα targeting. Their monoclonal antibody was licensed non-exclusively to Biocare Medical (Concord, CA) to enable manufacturing and distribution of IHC kits for research applications. The kit was developed in a manual use format, and for use on Biocare IntelliPath™, Dako Link and Leica Bond instruments. Studies evaluated the frequency and expression levels of FRα in multiple tumor types to enable decisions regarding tumor targets and histologic subtypes. Kits were developed in two formats, a multi-tumor kit and an ovarian tumor kit, to extend their use to multiple tumor types with a broad range of FR expression. Based on discussion with the US FDA, this assay is currently being validated for use in selecting patients for planned clinical trials.

Wettergren in *“The use of folate transporters as biomarkers in colorectal cancer therapy*” reported high expression of SLC46A1/PCFT, SLC19A1/RFC-1, and ABCC3/MRP3 was associated with longer 5-year disease-free survival (DFS) of patients with colorectal cancer (*n* = 363) treated with adjuvant 5-fluorouracil plus leucovorin (FLV), whereas there was no association between expression and DFS in untreated patients [53]. Furthermore, a positive association between 3-year progression-free survival and ABCC3/MRP3 expression was found in patients with advanced colorectal cancer (*n* = 294). She concluded that the folate transporter genes may have predictive value for patients with colorectal cancer going through FLV-based treatment.

Peters presented the *“Role of PCFT in PMX resistance of malignant mesothelioma: update of clinical evidence and new pharmacological tools”.* Here, he identified PCFT as a novel biomarker in malignant mesothelioma. In two cohorts totaling 124 patients treated with PMX and carboplatin, high expression (either protein or mRNA) of PCFT was associated with a longer overall survival (OS) of patients, while low TS expression was also associated with a longer survival. Furthermore, a combination of low PCFT/high TS resulted in very poor survival. Notably, RFC expression was not related to OS. He concluded that PCFT and TS can be considered predictive biomarkers for malignant mesothelioma and should be tested prospectively to select patients who would be most responsive to PMX-based therapies [54].

#### Chimeric antigen receptor cell therapies of cancer

Chimeric Antigen Receptor (CAR T) cell-based therapies have transformed pediatric oncology by producing high remission rates and durable responses in CD19+ B-cell malignancies. This scenario is ideal as CD19 expression is homogeneous and human blood provides a favorable environment for CAR T cells to thrive and destroy cancer cells (along with normal B cells). However, CAR T cell therapies for solid tumors remain a challenge due to problems with tumor heterogeneity, cytokine release syndrome (CRS) and CAR T cell exhaustion. Several talks reported exciting new approaches in this promising field.

**D. Powell** (University of Pennsylvania, USA) already presented in the previous edition of this meeting a FRα CAR T cells able to efficiently eliminate aggressive ovarian cancer xenografts in a murine subcutaneous tumor model [55]. Based on very promising safety and efficacy data, plans for a human clinical trial are underway (NCT03585764). This year his presentation *“CAR T anti FR beta”* described the development of a CAR T cell products targeting FRβ for the elimination of acute myeloid leukemia (AML) blasts and tumor-associated macrophages (TAMs) in the tumor microenvironment. In a review of earlier published results, he showed that FRβ-specific CAR T cells eradicated AML xenografts in mice [56] and summarized studies in which the same FRβ CAR T cells selectively eliminated immunosuppressive FRβ + TAMs, resulting in control of cancer progression in a syngeneic mouse model of an FRβ-negative ovarian cancer. Based on these results, a combination therapy that directly eliminates cancer cells together with a CAR T cell therapy that can eradicate the FRβ + TAMs should be highly synergistic.

**P. Low** (Purdue University, USA) in “*A universal CAR T cell technology that avoids most limitations of current therapies”* introduced a strategy for a universal CAR T therapy designed to avoid limitations known to compromise current CAR T cell therapies. These include: 1) toxicities arising from excessive release of cytokines (CRS); 2) resistance deriving from loss of the targeted tumor antigen; 3) exhaustion stemming from chronic exposure to tumor antigen; and 4) the need to engineer a different CAR T cell for each antigenically distinct tumor. Low described a CAR T cell technology that required only a single important modification. Briefly, he has engineered a CAR that contains the usual activation motifs (CD3ζ and 4-1BB), but expresses a scFv against fluorescein instead of the usual scFv against a tumor antigen. The resulting CAR T cell kills cancer cells and proliferates only when it encounters a cancer cell that labeled with fluorescein isothiocyanate (FITC). For the latter, they constructed a FITC-folate conjugate (bispecific adapter) designed to bridge between the anti-FITC CAR T cell and the FR-expressing cancer cell. Upon establishment of this bridge, the CAR T cell both proliferates and kills the cancer cell. He demonstrated that a variety of solid tumors in NOD SCID gamma mice could be eradicated upon addition of FITC-folate. Moreover, when symptoms of a CRS became severe, these could be suppressed either temporarily by interrupting adaptor administration, or by injecting excess fluorescein to block adaptor bridging. To prevent disease recurrence that can arise if a cancer cell mutates to delete the targeted antigen, he administered a cocktail of low molecular weight bispecific adapters, each comprised of fluorescein linked to a different tumor-specific ligand that recognized one of the remaining tumor antigens. Finally, to avoid CAR T cell exhaustion associated with chronic exposure of CAR T cells to antigen, he periodically deleted bispecific adapter addition.

**J. Lu** (Endocyte Inc., USA) in *“Preclinical Evaluation of Bispecific Adaptor Molecule Controlled Folate Receptor CAR T Cell Therapy”* presented a more detailed examination of the CAR T adaptor molecule platform. She found that maximal CAR T cell cytolytic activity correlated with: 1) the level of expression of FR on the cancer cell’s surface; 2) the number of CAR T cells that accumulated within the solid tumor; 3) the concentration of bispecific adapter (EC17, folate-fluorescein); 4) prevention of CAR T cell exhaustion; and 5) unidentified immunosuppressive factors within the tumor microenvironment. It was concluded that with proper optimization, this novel strategy could address several unmet needs in the CAR T therapy field.

#### Closing remarks

Altogether, the research presented at 7th meeting of the Folate Receptor Society highlighted the continued relevance of folate receptors/transporters in cancer and other diseases and the up-coming clinical exploitations of specific drugs and biological tools. In addition, the success of the 2018 meeting strengthens the Folate Receptor Society in promoting its important mission. These include: to foster sharing and discussion of the ongoing developments in the areas of folate receptors/transporters and one-carbon metabolism; and to organize every 2 years an international meeting in which distinguished and trainee scientists with different expertise can meet in an informal way to discuss current research challenges and to identify new avenues in basic and translational research. We thank the organizers, sponsors, and all the participants who made the 2018 meeting a tremendous success and we look forward to the 8th meeting of the Folate Receptor Society which will be held in Austin, Texas in 2020.

## Abbreviations

[^18^F]FLT: Fluorothymidine F^− 18^
ADC: Antibody drug conjugate
AICARFTase: 5-aminoimidazole-4-carboxamide ribonucleotide formyltransferase
AML: Acute myeloid leukemia
ATIC: inosine monophosphate cyclohydrolase
BBB: Blood-brain barrier
BLM: Bleomycin
C1: one-carbon
CAM: CAR T adaptor molecule
CAR: Chimeric Antigen Receptor
CFD: Cerebral Folate Deficiency
CIC: human Capicua gene
CRS: Cytokine release syndromes
CSF: Cerebral spinal fluid
DFS: Disease-free survival
DOX: Doxorubicin
FITC: Fluorescein isothiocyanate
FR: Folate receptor
GARFTase: Glycinamide ribonucleotide formyltransferase
GPI: Glycosylphosphatidyl inositol
IHC: Immunohistochemical
JAK: Janus Kinase
LRP2: Low Density Lipoprotein Receptor Related Protein 2
mAbs: Monoclonal antibodies
MDR: Multidrug Resistance
MTHFD: Methylene tetrahydrofolate dehydrogenase
MTHFD2L: homologous to MTHFD2
MTHFR: Methylenetetrahydrofolate reductase
MTX: Methotrexate
MTX-PG: Methotrexate polyglutamates
N4Py: N,N-bis (2-pyridylmethyl)-N-bis (2-pyridyl)-methylamine
NIR: Near-infrared
NMD: Nonsense-mediated mRNA decay
OS: Overall survival
PCFT: Proton-coupled folate transporter
Pgp: P-glycoprotein
PMX: Pemetrexed
PtdCho: Phosphatidylcholine
RA: Rheumatoid arthritis
RFC: Reduced folate carrier
SHMT: Serine hydroxymethyltransferase
TAA: Tumor Associated Antigen
TAMs: Tumor associated macrophages
TCT: targeted chemotherapy or alternatively targeted conventional therapy
THF: Tetrahydrofolate
TS: Thymidylate synthase
VDR: Vitamin D nuclear receptor
